# Supplementary material for: Understanding depression treatment and perinatal service preferences of Kenyan pregnant adolescents: A discrete choice experiment
Source: PLoS One. 2023 Mar 8;18(3):e0273274. doi: 10.1371/journal.pone.0273274 (PMC9994687; doi:10.1371/journal.pone.0273274)
Supplement: S1 File — (DOCX) [file pone.0273274.s001.docx]

**S1:** *Supplement Table 1* *Process checklist, indicators and key outcomes in developing DCE*

| **Process checklist** | **Process indicators** | **Outcomes** |
| --- | --- | --- |
| 1. Research Question | - This study measured the treatment preferences of pregnant adolescent girls aged 14-18 years - A DCE was used to measure trade-offs. | We received a lot of inputs form expert and community feedback on mental health needs of pregnant adolescents- we wanted a more empirically robust methodology to triangulate these findings to further adapt group interpersonal psychotherapy for depression to be delivered in primary care settings. These findings are valuable for common mental disorders in general and for tailored mental health service development. |
| 1. Attributes and Levels | - Attributes and levels were identified using mixed methods. - Qualitative methods included evidence synthesis, expert consultation, stakeholder engagement, and pretest interviews. - Quantitative methods included pilot testing | We have published several studies covering these aspects [1–3]  We piloted using 10 participants to refine the experiment. |
| 1. Construction of tasks | - Example task was provided. - Full-profile, paired-comparison choice tasks (eight attributes per profile, two profiles per task). - Opt-out was included | The tasks were developed keeping the study findings as well as review and comments from key stakeholders |
| 1. Experimental Design | - This study used a blocked D-efficient Bayesian design. - Balance was achieved. - 10 choice tasks were found to be appropriate during pilot testing | D-optimal designs maximize the precision of the estimated parameters given a set number of choice tasks and information on expectations of respondent preferences .[4] |
| 1. Preference elicitation | - The method enables measurements of trade-offs. - We did not allow indifference. - Explanations about how to complete the task and general orientation of data collectors and participants on ordering of relative importance of the attributes were added. | Participants were allowed to choose between two options. We allowed the participants to select an opt-out option  [5]. |
| 1. Instrument Design | - Demographic and other characteristics were collected. - Descriptions on attributes, levels, and decision context were included. - Response burden for the survey was assessed during pretesting and pilot testing | Pre-testing and multiple feedback rounds testing why participants gave specific responses were important to establish relevance of the attributes and levels. |
| 1. Data Collection | - Purposive sampling- only pregnant adolescents of ages 14-18 - Continuous recruitment - Interviewer administered via tablet - Ethical approval was obtained | Though the data was collected during the pandemic, there was seamless data gathering process and we recruited community health workers who used the tablets that had DCE embedded within Dooblo program |
| 1. Statistical Analysis | - Analysis used mixed logit to model preference heterogeneity | The mixed logit (MXL) model overcomes some of the limitations of the MNL model. MXL estimation accommodates unobserved taste heterogeneity by specifying preference parameters as random variables with means and standard deviations rather than fixed parameters. MXL involves three main specification issues: (1) determination of the parameters that are to be modelled as random variables; (2) choice of so-called mixing distributions for the random coefficients; and (3) economic interpretation of estimated random coefficients. |
| 1. Results and Conclusions | - Key preferences revealed were longer therapy duration, therapy delivered by nurses, training in parenting skills, combined refreshments to facilitate mental health treatment and a negative preference for MCH services with older adult women were revealed - We think being sensitive to negative preferences (disutility) will be important | These preferences reveal some of the communication, logistic and cultural needs that need addressing for engaged mental health treatment. |

**References:**

^1 Kumar M, Huang K-Y, Othieno C,^ *^et al.^* ^Adolescent Pregnancy and Challenges in Kenyan Context: Perspectives from Multiple Community Stakeholders.^ *^Glob Soc Welf^* ^2018;^**^5^**^:11–27. doi:10.1007/s40609-017-0102-8^

^2 Kumar M, Chu W, Gellatly R,^ *^et al.^* ^Identified needs of peripartum adolescents in Sub-Saharan Africa from 2013 to 2021: a mapping of domains for strengthening psychosocial interventions.^ *^Curr Opin Psychol^* ^2022;^**^45^**^:101291. doi:10.1016/J.COPSYC.2021.12.003^

^3 Yator O, Khasakhala LI, John-Stewart G,^ *^et al.^* ^Acceptability and Feasibility of Group Interpersonal Therapy (IPT-G) for Depressed HIV+ postpartum adolescents delivered by community health workers: a protocol Paper.^ *^Clin Med Insights Psychiatry^* ^2020;^**^11^**^:1179557320951222.^

^4 Jonker MF, Bliemer MCJ. On the Optimization of Bayesian D-Efficient Discrete Choice Experiment Designs for the Estimation of QALY Tariffs That Are Corrected for Nonlinear Time Preferences.^ *^Value Heal^* ^2019;^**^22^**^:1162–9. doi:https://doi.org/10.1016/j.jval.2019.05.014^

^5 Bridges JFP. Stated preference methods in health care evaluation: an emerging methodological paradigm in health economics.^ *^Appl Health Econ Health Policy^* ^2003;^**^2^**^:213–24.^
