# Supplementary material for: Understanding depression treatment and perinatal service preferences of Kenyan pregnant adolescents: A discrete choice experiment
Source: PLoS One. 2023 Mar 8;18(3):e0273274. doi: 10.1371/journal.pone.0273274 (PMC9994687; doi:10.1371/journal.pone.0273274)
Supplement: S2 File — (DOCX) [file pone.0273274.s002.docx]

## **S2 Mixed logit model**

Choice experiment modelling is based on random utility theory (RUT) which assumes that the utility (U) for individual i conditional on choice j consists of an explainable component (Vij) and a random component (eij) (formula 1). The random component may capture any combination of unobserved attributes, unobserved preference variation, specification error, measurement error and inherent variability within and between individuals (6).

1. *Uij =Vij + eij*

For this analysis we applied dummy coding. For our main effects final model, we selected a mixed logit regression model to account for preference heterogeneity with all attributes included as random parameters. The explainable component (Vij) for this experiment is denoted in formula 2 below, where b1-9 represents the coefficient for the corresponding attribute level. The baseline attribute category for each attribute is omitted from formulae and estimations, as this attribute has by definition a utility of 0 when dummy coding is used.

## Vij = b1 Information Delivery: Individualized dietary information + b2 Joint Participation: Provide information sheets for care-givers + b3 Treatment duration: 8 sessions for 1.5 hours + b4 Intervention delivery agents: Facility Nurses + b5 Further Training Needs: Formal (Back to school) + b6 Support Type: Parenting skills + b7MCH Services: Combined with adult/older women + b8 Incentives: Food+ b9 Incentives: Transport.

Mixed logit models were fit using R’s version 4.1.2 mlogit command which uses simulated maximum likelihood estimators and generates mean utilities for the population and standard deviations of the random coefficients. Mixed logit coefficients (*b*) can be interpreted as the strength of the relative preference for the particular attribute comparison, with positive coefficients representing positive preferences (desirable) and negative coefficients representing negative preferences (less desirable). Standard deviations represent preference heterogeneity for attribute comparisons, with a 0 standard deviation indicating no heterogeneity.
