# Supplementary material for: Understanding depression treatment and perinatal service preferences of Kenyan pregnant adolescents: A discrete choice experiment
Source: PLoS One. 2023 Mar 8;18(3):e0273274. doi: 10.1371/journal.pone.0273274 (PMC9994687; doi:10.1371/journal.pone.0273274)
Supplement: Attachment — Submitted filename: A discrete choice experiment to understand depression intervention treatment preferences of Kenyan pregnant adolescents.docx [file pone.0273274.s004.docx]

A discrete choice experiment to understand depression intervention treatment preferences of Kenyan pregnant adolescents

This is a novel research topic on a very important issue. Mental health is increasingly highlighted as a global concern, as are adolescents who are pregnant or parents in LMICs. There is very little research which engages young people on preferences for treatment and support – and this is excellent to see.

I would recommend this for publication, with some suggested edits:

1. Some of the wishes of participants go beyond depression intervention treatment – for example the desire for specialised ANC services distinct from adult services. Perhaps this could be reflected a bit more in the title and abstract? It is certainly coming into the discussion.
2. I would add into the introduction that there has been a lack of research that includes and asks pregnant adolescents what they would want from such services – at least quantitatively – and cite any other studies that attempt this. A bit of review of existing evidence on adolescent preferences for mental health services in this introduction stage would be helpful.
3. Nice clear description of methods. Excellent way of compiling the list of choices, including qualitative and pilot stages. Very robust.
4. A bit more information about the purposive sampling would be useful here. In what ways was it purposive? Were all registered adolescents approached or was there purposive sub-sampling within this group?
5. Stable mental health was a criteria – please unpack this a bit more – what was meant by this?
6. Results very clearly and well written up.
7. Discussion – starts with ‘consistent with prior expectations’ – is this based on literature review of prior studies of preferences? Again this would be helpful to include.
8. The limitations are mentioned in two separate paragraphs apart from each other – it would help the flow to move them together
9. Use of this study to modify and improve group IPT is mentioned – it would be good to include this context in the introduction
10. There are some parts of the discussion which may be valuable to think about including in the introduction rather – for example setting context on patient involvement.

Overall an excellent and valuable study. It would benefit from a bit of restructuring between discussion and introduction, and some literature review of existing work – but these should be easily achieved. Well done to the author and team.
